# Clinicopathological analysis of HOXD4 expression in diffuse gliomas and its correlation with *IDH* mutations and 1p/19q co-deletion

**DOI:** 10.18632/oncotarget.23371

**Published:** 2017-12-18

**Authors:** Xin-Wei Zhao, Yun-Bo Zhan, Jian-Ji Bao, Jin-Qiao Zhou, Feng-Jiang Zhang, Yu Bin, Ya-Hui Bai, Yan-Min Wang, Zhen-Yu Zhang, Xian-Zhi Liu

**Affiliations:** ^1^ Department of Neurosurgery, The First Affiliated Hospital of Zhengzhou University, Zhengzhou, Henan 450001, China

**Keywords:** gliomas, HOXD4, immunohistochemistry, TCGA, prognosis

## Abstract

**Backgrounds:**

HOX (homologous box) is known as the dominant gene of vertebrate growth and cell differentiation. Abnormal expression of HOX gene in various tumors has attracted the attention of scholars. As a component of HOX clusters, HOXD4 plays a controversial role in the tumorigenesis of central nervous system.

**Results:**

The data demonstrated that and the results demonstrated that HOXD4 was overexpressed in glioma tissues compared to that of normal brain tissues. patients with high HOXD4 expression had a significant shorter survival than those with low HOXD4 expression in total glioma cohort (*p*<0.001), WHO Grade II cohort (*p*=0.003) and Grade III cohort (*p*<0.001), but not in Grade IV cohort when OS (overall survival) was analyzed (*p*=0.216). The findings were confirmed by the large-scale omics data analysis including lower-grade glioma (LGG) and glioblastoma multiforme (GBM) in TCGA (the cancer genome atlas) and CGGA (Chinese glioma genome atlas). Moreover, it was revealed that the expression of HOXD4 have a significant impact on the OS of Grade IV glioma with *IDH* wild-type and 1p/19q intact according to TCGA data.

**Methods:**

Clinicopathological analysis of HOXD4 expression in 453 glioma patients was performed in the current study. Expression of HOXD4 was evaluated by qPCR and immunohistochemical (IHC) staining. Univariate and multivariate analysis were conducted to investigate the prognostic role of HOXD4 in glioma patients.

**Conclusions:**

Expression of HOXD4 was closely related to the clinical outcomes of patients with gliomas, and HOXD4 may be a potential prognostic biomarker of gliomas.

## INTRODUCTION

Glioma is recognized as the most common malignant neoplasm occurred in the central nervous system [1]. Gliomas ranked the first in the incidence of intracranial tumors, and were characterized by high aggressiveness, recurrence rate and mortality [2]. According to the WHO classification, gliomas are divided into four degrees [3]. There are many hypotheses about the pathogenesis of glioma, such as genetic factors, radiation exposure and nitrite contact [4]. At present, the treatments of gliomas include surgical resection with postoperative comprehensive treatment (radiotherapy, chemotherapy and gene targeting therapy). Nevertheless, the survival times of glioma patients are not significantly prolonged, especially in glioblastoma patients, whose median survival time is only 12-14 months [5–11]. Due to highly invasive nature of gliomas, postoperative treatments cannot prevent tumor recurrence [12]. Meanwhile, side effects of radiotherapy and chemotherapy on patients make more harm than good [13]. To stratify and improve the prognosis of glioma patients, it is increasingly important to identify more potential biomarkers and therapeutic targets.

HOX gene, which is also known as homologous box gene, has a close relationship with the early embryo development [14–16]. As the main gene of vertebrate growth and cell differentiation, it plays an important role in the development of central nervous system, axial bone, gastrointestinal tract, incontinence, external genitalia and limb [17, 18]. A total of 39 homologous box sequences were found in the mice and humans, which could be divided into 13 groups according to the position on the chromosome [19]. In the previous study, the HOX gene was overexpressed by investigating the spontaneously derived tumor-bearing canine breast cancer model. The expression profile was consistent with the oncogene-like features (HOXA1, HOXA13, HOXD4, HOXD9 and SIX1) [20]. It was also reported that HOX cluster genes promoted the proliferation and differentiation of neuroblastoma cells [21]. But the impact of HOXD cluster genes in glioma has not been clarified up to now.

In the current study, we identified the up-regulated expression of HOXD4 in diffuse glioma tissues. Meanwhile, expression of HOXD4 was revealed an independent prognostic factor in patients with gliomas. Furthermore, data acquired from TCGA and CGGA were also analyzed to corroborate our findings.

## RESULTS

### Expression of HOXD4 was elevated in gliomas and public database (TCGA and CGGA)

**Figure 1:**
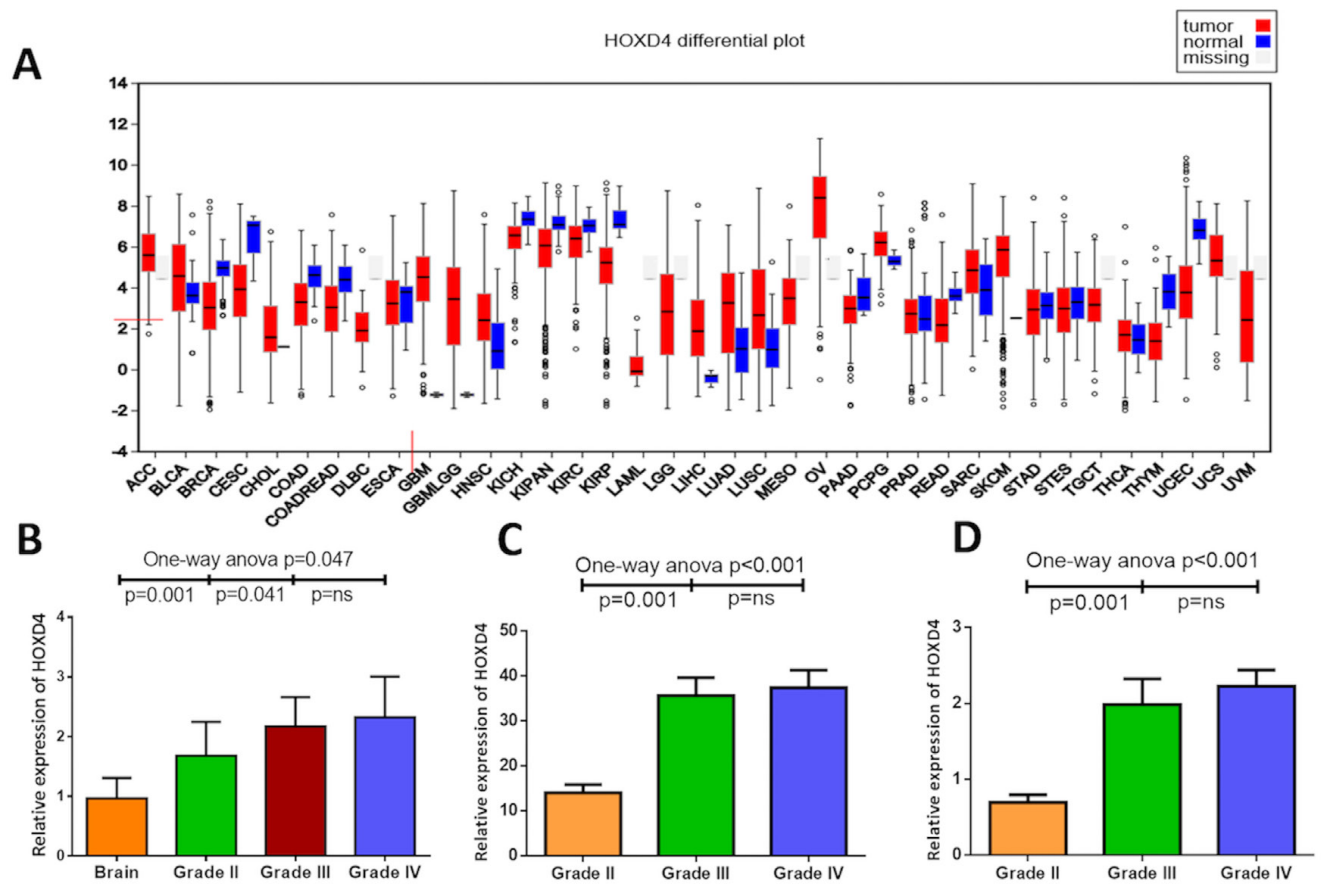
Abnormal expression of HOXD4 in diffuse glioma at mRNA level **(A)** HOXD4 expression at FireBrowse (http://firebrowse.org/viewGene.html?gene=HOXD4). **(B)** Relative quantification (RQ) values of HOXD4 by PCR showed a higher HOXD4 expression in Grade II glioma than brain (*p*=0.001), and higher in Grade III compared with II (*p*=0.041) while not significant between Grade III and IV (*p*=ns). **(C)** Reads per kilobase per million (RPKM) of HOXD4 from TCGA was calculated (*p*=0.001), significantly higher in Grade III glioma than II (*p*=0.001), not in Grade IV than III (*p*=ns). **(D)** Log_2_ (RPKM+1) of HOXD4 was calculated from CGGA (*p*=0.001), significantly higher in Grade III glioma than II (*p*=0.001), not in Grade IV than III (*p*=ns).

To investigate the expression of HOXD4, qPCR was performed in a panel of 44 glioma tissues including 23 primary GBMs (Grade IV), 10 AAs (Grade III) and 11 OAs (Grade II), as well as 10 non-neoplastic brain tissues for control. As results was shown (Figure 1B), One-way Anova analysis with Bonferroni correction demonstrated that the expression of HOXD4 was remarkably elevated in gliomas than normal brain (*p*=0.047). Meanwhile, the expression of HOXD4 is significantly higher in GBM than LGG as well (*p*=0.004).

**Figure 2:**
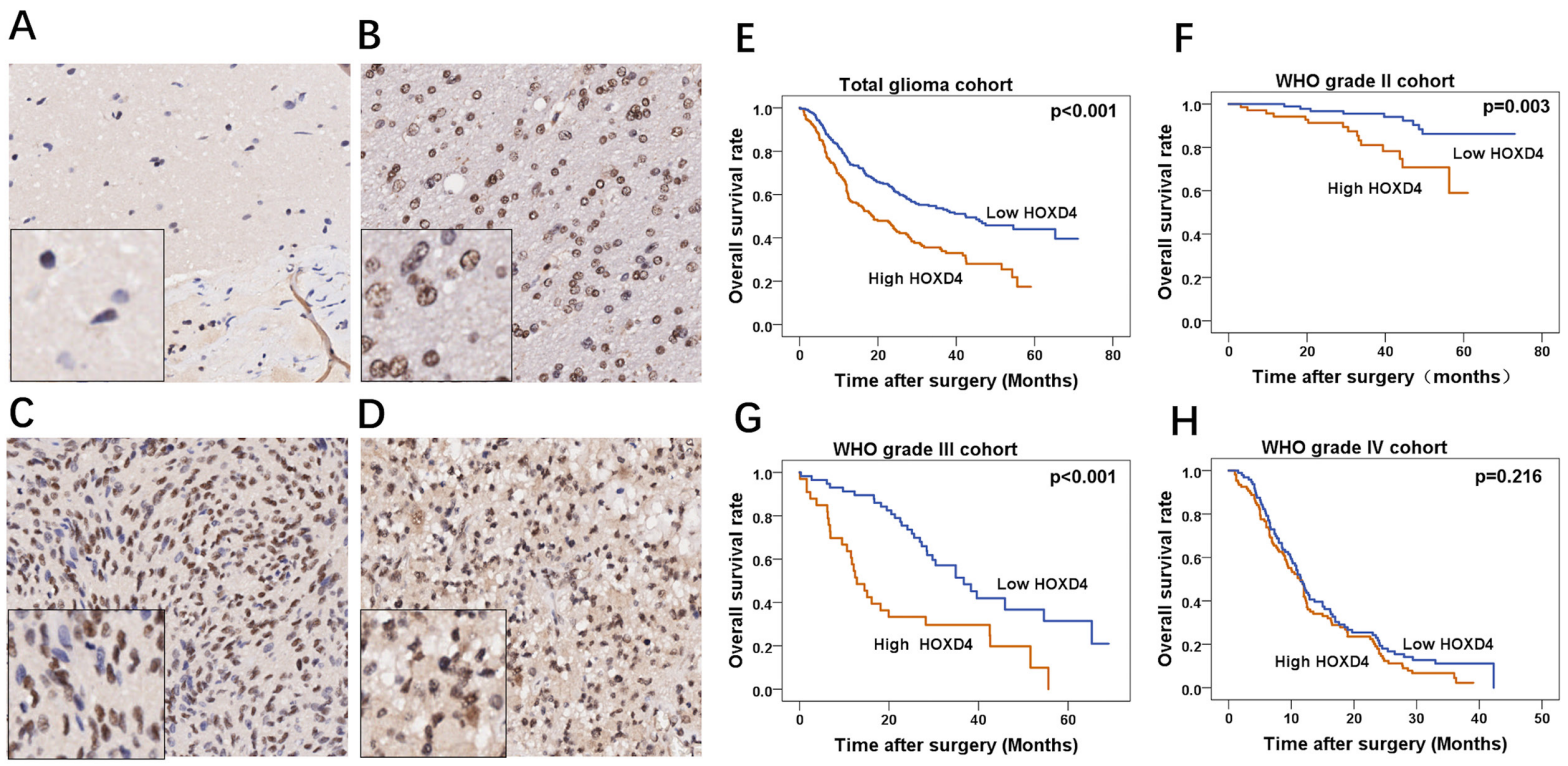
Immunohistochemical staining of HOXD4 in glioma and survival analysis of 453 patients by Kaplan-Meier method **(A-D)** FFPE tissues with HOXD4 expression of normal brain, gliomas of WHO grade II, III and IV respectively (×200, scale bars 50μm), the left bottom of the picture is the enlarged version (×100). **(E-H)** Overall survival curve by Kaplan-Meier method in cohorts of total glioma patients (*p*<0.001), glioma WHO grade II (*p*=0.003), III (*p*<0.001) and IV (*p*=0.216) respectively.

To further confirm our revealings, RNA sequences of TCGA and CGGA were acquired and analyzed to identify the HOXD4 expression and its prognostic role in gliomas. Bioconductor/TCGA biolinks function package from TCGA (https://tcga-data.nci.nih.gov/tcga/) was used to download and pretreat GBM and LGG mRNA expression RNASEqV2 data. Expression-logs of HOXD4 of gliomas in mRNA level was analyzed by One-way Anova method, and results demonstrated that the expression of HOXD4 was significantly higher in WHO grade IV gliomas than Grade IIIgliomas (*p*<0.001 Figure 1C). The expression of HOXD4 was considerably higher in WHO grade III gliomas than Grade II gliomas (*p*<0.001). Similarly, we conducted One-way Anova analysis to analyze the CGGA data. The expression of HOXD4 in glioma tissues was ladder-like elevated as the pathological grades escalated (Figure 1D), which is in accordance with the results of TCGA data analysis. The staining was localized mainly in the nucleus, with a small amount of cytoplasm. High expression of HOXD4 was determined in 178 samples, consisting of 39.29% in all glioma samples while the others were considered as low expression (60.71%). We calculated the average values of the staining scores of HOXD4 expression in normal brain tissues and gliomas. The staining scores corresponding to brain tissue and Grade II, III, IV gliomas was 0.5±0.14, 2.1±0.55, 3.3±0.63, 4.8±0.59 respectively (Figure 2A-2D). One-way Anova analysis with Bonferroni correction revealed that staining score of HOXD4 was higher in glioma than normal brain (*p*=0.021), and also significantly higher in GBM than LGG (*p*=0.001).

### HOXD4 was revealed as an independent prognostic factor in glioma patients

A total of 527 patients were followed up from 2011 to 2014 at the First Affiliated Hospital of Zhengzhou University, and clinical data of 453 patients were collected, missing rate was 14.1%, the median durational follow-up time was 25.9 months. The deadline of follow-up was 2017.8.30. Kaplan-Meier survival curve was used to demonstrate the relationship between HOXD4 expression and survival times of glioma patients. Results showed that in total cohort, patients with high HOXD4 expression have a significantly worse OS and PFS than those with low HOXD4 expression (Figure 2E *p*<0.001). Meanwhile, in different grades of gliomas, univariate analysis demonstrated that patients with high HOXD4 expression had a considerably shorter OS than that with low HOXD4 expression in Grade II (Figure 2F *p*=0.003) and Grade III glioma cohorts (Figure 2G *p*<0.001), but not in Grade IV gliomas (Figure 2H *p*=0.216). The clinicopathological features of the glioma cohort was summarized according to the HOXD4 expression level (Table 1). HOXD4 IHC scores was high in 219 samples and low in 234 samples according to the cut-off score by ROC curve. There was significant association between HOXD4 IHC score and patient age, recurrence, or WHO Grades (*p*> 0.05 for all covariates). Univariate analysis demonstrated that patients age (*p*<0.001), extent of resection (p=0.012), postoperative radiation therapy (*p*<0.001), postoperative chemotherapy (*p*<0.001) and HOXD4 expression (*p*<0.001) impact the prognosis in total glioma patients. In multivariate survival analysis, we included prognostic factors with significance such as complete resection, radiotherapy, chemotherapy, Karnofsky Performance Status (KPS score) and expression of HOXD4. Data analysis demonstrated that HOXD4 expression was an independent prognostic factor significantly influencing the survival of patients with gliomas (Table 2).

**Table 1:** Association between HOXD4 expression by IHC and clinicopathological features of 453 glioma patients

Factors	No. of cases	High HOXD4 n =219	Low HOXD4 n =234	P-value
Sex				
Male	256	123	133	0.885
Female	197	96	101	
Age				
≤40	67	35	32	**0.010**
>40	386	184	202	
^*^KPS				
≤80	148	74	74	0.623
>80	305	145	160	
Extent of resection				
Gross total	343	150	193	**0.001**
Subtotal	110	69	41	
Recurrence				
Yes	282	154	128	**0.002**
No	171	65	106	
Grade				
II	160	70	90	**0.013**
III	90	35	55	
IV	203	113	90	
Histology				
Astrocytoma	85	35	50	0.086
Oligodendroglioma	165	81	85	
Glioblastoma	203	112	91	

The test statistic is Fisher’s exact test; two tailed P value.

**Table 2:** Multivariate Cox Proportional-Hazards Models for gliomas

Factors	OS		PFS	
	OR (95% CI)	*p* value	OR (95% CI)	*p* value
KPS^a^(≤80 or >80)	1.224 (0.94-1.60)	0.136	1.15 (0.89-1.49)	0.259
Complete resection (yes or no)	1.290 (0.97-1.71)	0.075	1.28 (0.98-1.68)	0.071
RT^b^ (yes or no)	2.040 (1.48-2.88)	**0.001**	1.91 (1.37-2.68)	**0.001**
CHT^c^ (yes or no)	1.148 (0.82-1.60)	0.411	1.07 (0.76-1.47)	0.688
HOXD4 expression (high or low)	0.468 (0.36-0.60)	**0.001**	0.53 (0.42-0.68)	**0.001**

Bold values indicate *p*<0.05.

OR odds ratio, CI confidence interval.

^a^ Indicating Karnofsky Performance Status.

^b^ Indicating postoperative primary radiation therapy.

^c^ Indicating postoperative primary chemotherapy.

### The expression of HOXD4 influence the prognosis of glioma patients according to the data from TCGA and CGGA

**Figure 3:**
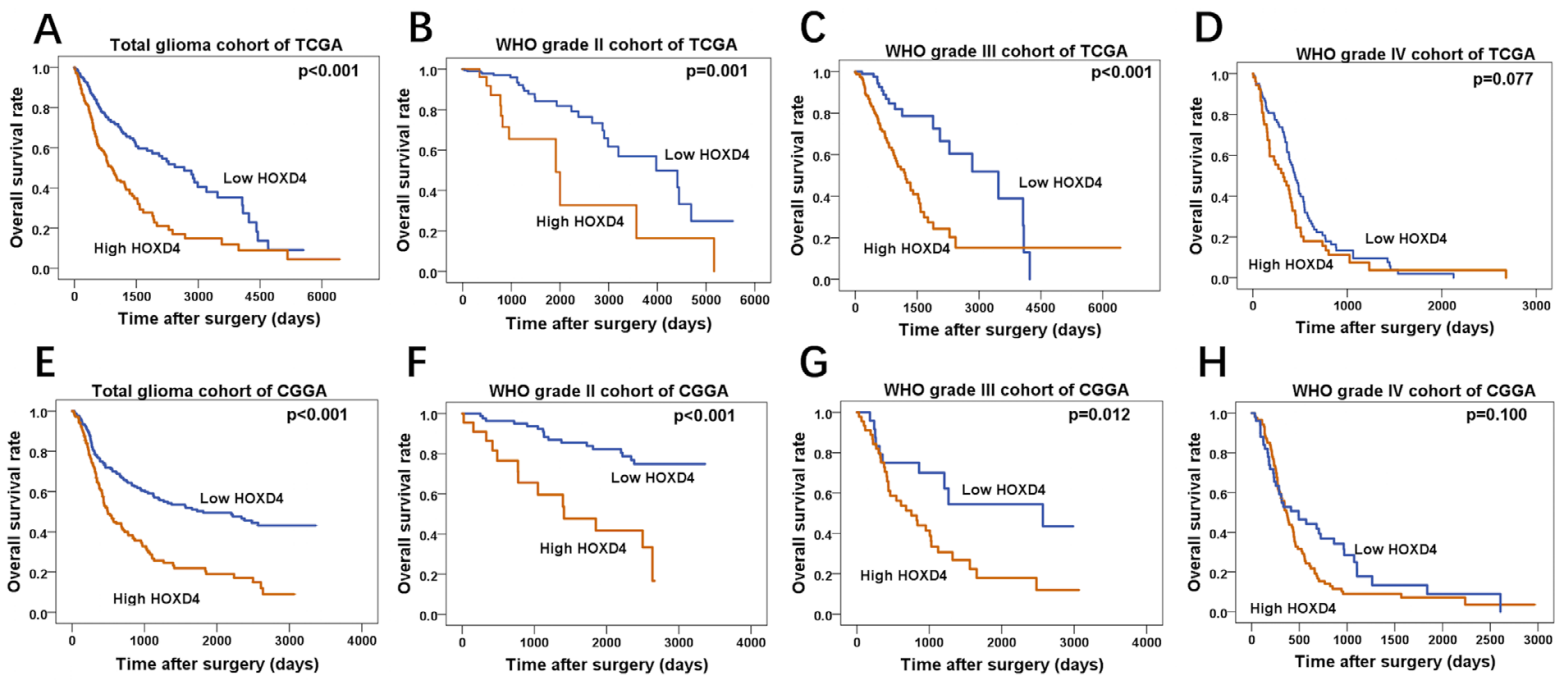
Survival analysis of TCGA and CGGA glioma cases **(A-D)** Overall survival curve by Kaplan-Meier method for cohorts of TCGA, High HOXD4 expression cause a shorter survival period of patients in total glioma (*p*<0.001) and WHO II (*p*=0.001), III (*p*<0.001) cohorts than low HOXD4, while there is no significant correlation in WHO IV glioma (*p*=0.077). **(E-H)** Overall survival curve by Kaplan-Meier method for cohorts of CGGA, patients with high HOXD4 have an unfavorable prognosis in total glioma (*p*<0.001) and WHO II (*p*<0.001), III (*p*=0.012) cohorts than low HOXD4, WHO IV glioma cohorts show no difference (*p*=0.100).

By the matching of barcode each sample allocated by TCGA, in 595 LGG and 165 GBM patients, we mapped each patient–s clinical data (including OS) to its RNA sequence. These cases were divided into group II, III and IV according to the WHO grades as well. In each cohort, the cases were separated into high HOXD4 expression group and low HOXD4 expression group according to expression-log of HOXD4. As the Kaplan-Meier survival curve revealed, in total glioma patients (Figure 3A *p*<0.001) and WHO II (Figure 3B *p*=0.001), III (Figure 3C *p*<0.001), patients with high HOXD4 expression have a significantly shorter OS than those with low HOXD4 expression. However, HOXD4 expression is not a prognostic factor of significance in Grade IV gliomas (Figure 3D *p*=0.077). The cases from CGGA included 181 LGG and 144 GBM patients with the matching RNA sequence. Univariate analysis demonstrated that group with low HOXD4 expression had a considerably better OS than group of high HOXD4 expression in total glioma patients (Figure 3E *p*<0.001) and WHO II (Figure 3F *p*<0.001), III (Figure 3G *p*=0.012). There is no statistical significance between HOXD4 expression and survival time in Grade IV glioma cohort (Figure 3H *p*=0.100).

### HOXD4 expression influences the OS in GBM patients with *IDH* wild-type and 1p/19q intact

**Figure 4:**
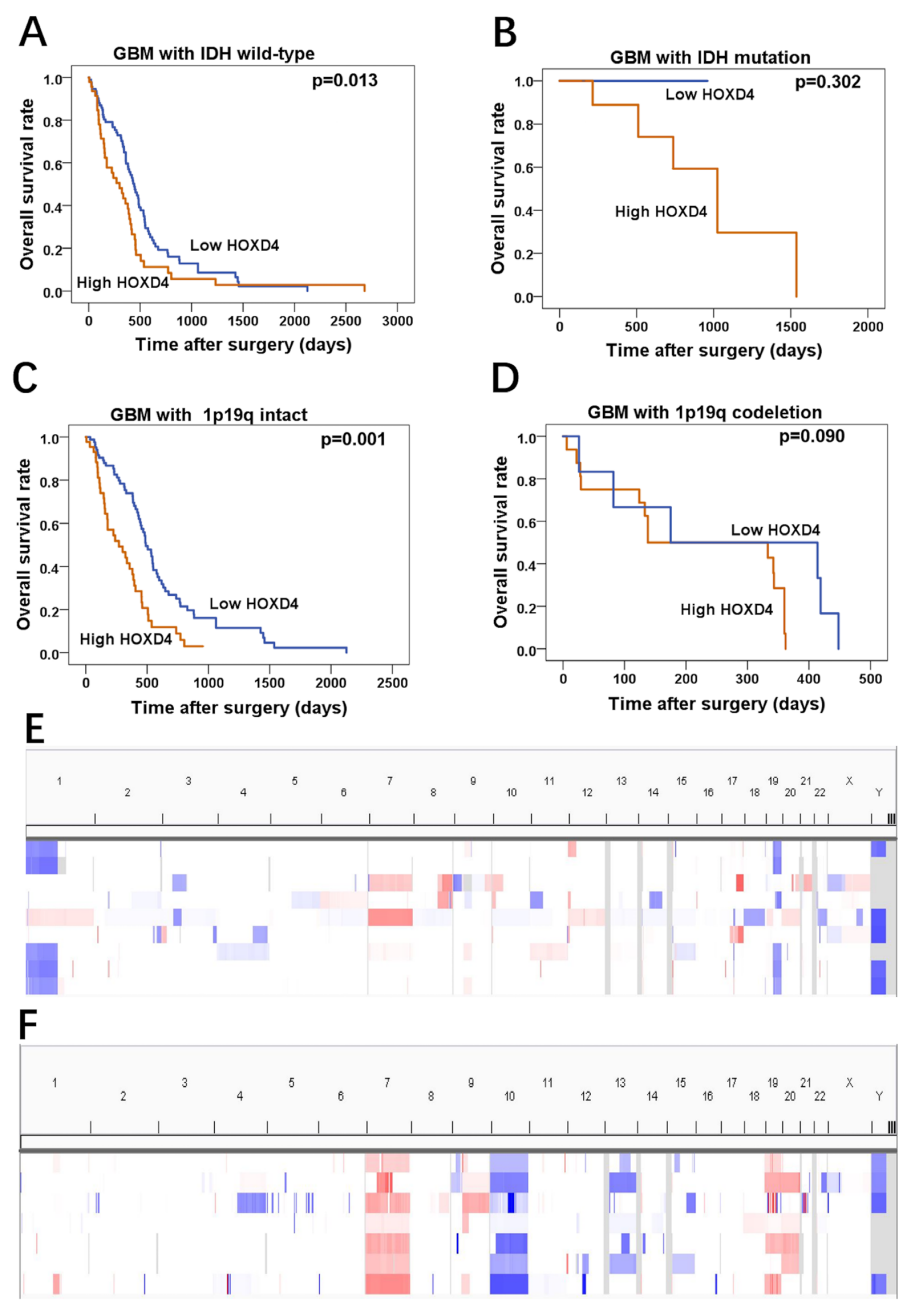
HOXD4 expression impact the OS of GBM patients with *IDH* wild-type or 1p19q intact by TCGA data analysis **(A-B)** Prognosis of GBM with *IDH* wild-type was associated with HOXD4 expression (*p*=0.013), but prognosis of GBM with *IDH* mutation was not significantly associated with HOXD4 expression (*p*=0.302). **(C-D)** Prognosis of GBM with 1p19q intact was associated with HOXD4 expression (*p*=0.001), but prognosis of GBM with 1p19q codeletion was not significantly associated with HOXD4 expression (*p*=0.090). **(E-F)**Analysis of Copy Number Variations (CNVs) in LGG and GBM samples from TCGA, respectively. Rows represent glioma patients and columns represent chromosomes. The colors in the heatmap indicate allelic states: white =normal heterozygous state; light red =imbalanced gain; Dark red =amplified heterozygous states; Light blue = copy neutral LOH and amplified LOH; Dark blue = LOH or physical loss in the context of a diploid genome.

In a further investigation of TCGA data, we also seek the gene mutation and chromosome gene copy number variation of glioma cohort, and then acquired *IDH* mutations and 1p19q co-deletion in these samples. In *IDH* wild-type subgroup of GBM, it was revealed that patients with high HOXD4 expression had significantly worse OS than that of patients with low HOXD4 expression (Figure 4A *p*=0.013), while this phenomenon was not observed in GBM patients with *IDH* mutation (Figure 4B *p*=0.302). Moreover, analysis of Copy Number Variations (CNVs) in LGG and GBM samples from TCGA was conducted by Integrative Genomics Viewer (Figure 4E, 4F). in survival analysis demonstrated that HOXD4 expression were a remarkable prognostic factor in 1p/19q intact subgroup of GBM (Figure 4C *p*=0.001), but not in 1p/19q co-deletion subgroup (Figure 4D *p*=0.090).

## DISCUSSION

In the current study, the results of PCR and immunohistochemical staining demonstrated that HOXD4 overexpression significantly correlated with the malignancy of gliomas and had a remarkable impact on the prognosis of glioma patients, and the revealing were also confirmed by statistical analysis from data of TCGA and CGGA.

HOX, short for homeo box, is named by its distinctive sequence which contains 180-183 bps [23]. 60-61 amino acids encoded by this sequence form a polypeptide region, which is known as the homeodomain. Expression of downstream target gene was regulated by the combination of homeodomain and specific DNA [14]. Studies have shown that HOX gene clusters played an important role in the progression in a variety of tumors. In the leukemia characterized by BCR/ABL and MLL fusion gene, the expression of HOX was elevated. Further studies revealed that HOX was a downstream target of MLL and HOX fusion gene, and involved in the formation of myeloid phenotype [24]. Expression of HOX clusters showed a dramatic inhibition caused by miRNA-10a in breast cancer, suggesting that they may play a functional role in the process of breast cancer [25]. In glioma development and progression, there are quite a few reports about the functions of HOX clusters. Previous studies found that HOXA9 in human gliomas promoted cell proliferation, and inhibited the apoptosis by suppressing PI3K-AKT pathway, which contributed to the development of tumors [26]. Besides, HOXA13 is a diagnostic marker for GBM and activated Wnt/TGF-β to promote glioma development [27]. Analysis of HOXD gene expression in human low-grade gliomas tissues revealed that HOXD1 and HOXD12 were highly expressed in gliomas, whereas the expression of HOXD3 was depressed [28]. It was also found that HOXD9 could promote the proliferation of glioma cells and inhibit cell apoptosis, the research identified the high expression of HOXD9 functioned as an enriched-cell fraction of glioma cancer stem-like cells [29]. In summary, the HOX gene is strongly associated with the pathogenesis of gliomas. and it should be emphasized that, when comparing with HOX gene research in lung cancer, leukemia and other tumors in the study, HOX gene still need deeper study for the molecular treatment of glioma [30].

In our current study, the expression of HOXD4 in Grade II, III, IV gliomas and normal brain tissue samples was detected by qRT-PCR, indicating that HOXD4 is widely present in glioma tissues. However, in the comparison of tumor tissue and normal brain tissue, we found that HOXD4 expression was significantly increased, suggesting that HOXD4 may play a role in the progression of gliomas. Multivariate survival analysis including putative prognostic factors such as KPS [31, 32], radiation therapy, chemotherapy, resection extent [33] demonstrated that HOXD4 was an independent prognosis factor in glioma patients. Furthermore, we analyzed the expression and survival data of TCGA and CGGA cohort concerning LGG and GBM on HOXD4, and revealed that HOXD4 expression was significantly increased in GBM patients compared to LGG patients. These data from the databases above further validated the preliminary results on HOXD4 derived from the data of our cohort.

In recent years, discovery of *IDH* mutations is one of the most important findings in glioma genomics. *IDH* mutations have a definite relationship with the survival of glioma patients, which has been widely recognized [34]. As *IDH* has a clear impact to the prognosis of glioma patients, survival analysis of GBM patients with *IDH* wild-type may be another solution for studying the effects of HOXD4 on clinical outcomes [35]. And *IDH*-wildtype glioblastoma could be defined clinically as primary glioblastoma [36]. Therefore, we further divided LGG and GBM cohorts of TCGA into different subgroups according to *IDH* mutation. It was hypothesized that HOXD4 could be considered as a potential predictor of GBM prognosis. For the similar reasons, GBM patients of 1p/19q intact was established as another model for the research of HOXD4 on the prognosis. And it was identified that HOXD4 was a potential predictor in GBM patients with intact 1p/19q chromosome.

There were also several limitations in the current research. First, although the sample size of our cohort was quite large and the conclusion was corroborated by enormous scale of samples in TCGA and CGGA database, the nature of the study is retrospective. Further prospective studies were needed to confirm our findings. Second, further experiments focused on molecular mechanism of HOXD4 in glioma carcinogenesis and progression was needed in the future.

In conclusion, the current results demonstrated the expression of HOXD4 was elevated in gliomas and closely correlated with the malignancy of gliomas. Moreover, HOXD4 was revealed as a potential prognostic factor in a large cohort by univariate and multivariate analysis. HOXD4 may be an important prognostic factor and a potential therapeutic target for glioma in the future.

## MATERIALS AND METHODS

### Patients, specimens and clinical data

The research was approved by the Human Scientific Ethics Committee of Zhengzhou University. 453 patients received surgery between November 2011 and December 2015 in The First Affiliated Hospital of Zhengzhou University were included in the current research. All cases were stained with hematoxylin & eosin (H&E) and centrally reviewed according to the 2007 World Health Organization (WHO) criteria [22]. Clinical characteristics of patients were retrieved from medical documents. The follow-up data was obtained by telephone-calls or out-patient clinic. Formalin-fixed paraffin-embedded (FFPE) tissues of primary tumor were collected simultaneously. Fresh-frozen glioma tissues including 11 cases of anaplantic astrocytomas (AAs), 10 cases of anaplastic oligodendrogliomas (OAs), 23 cases of GBMs and 10 normal brain tissues (collected in brain trauma surgery) were gathered in The First Affiliated Hospital of Zhengzhou university between August 2016 and November 2016. All patients had signed informed consents before tissue collection.

### TCGA and CGGA data analysis

Information of 595 LGG cases and 165 GBM cases was collected from TCGA. The data files included: (1) RNA sequencing; (2) DNA copy-number and single-nucleotide polymorphism arrays; (3) CNV-DNA copy; (4) Clinical data of patients. OS and PFS were computed by using the Kaplan-Meier survival curves and compared by the log-rank test. The cases were separated by WHO classification and then analyzed. The cut-off scores were calculated by the X-tile software (http://medicine.yale.edu/lab/rimm/research/software.aspx).

Data was explored through the following online data libraries:

the Broad Institute FireBrowse portal (http://firebrowse.org/?cohort=GBMLGG),

the cBioPortal for Cancer Genomics (http://www.cbioportal.org/cancerid=lgggbm_tcga_pub),

the Cancer Genome Atlas (https://cancergenome.nih.gov/),

the Chinese Glioma Genome Atlas (http://www.cgga.org.cn/),

the TCGA publication page (https://tcga-data.nci.nih.gov/docs/publications/lgggbm_2015/).

### Real-time quantitative PCR

Fresh-frozen glioma tissues was moved out from −80°C and grinded into tissue suspension, Trizol (Invitrogen, Carlsbad, US) was applied for extracting the RNAs of total cells, then the cDNA reverse transcription is accomplished by the using of RT Primer Mix and PrimeScript RT Enzyme Mix 1 (Takara, Japan), reaction of reverse transcription included 50ng of RNA, 4ul of 5×PrimeScript Buffer, 2ul of PrimeScript RT Enzyme Mix, The reaction was incubated for 15 min at 42°C, qRT-PCR was made by SYBR Premix Ex Taq (Takara, Japan), QRT-PCR was performed according to the SYBR® Premix Ex Taq ™ II (Tli RNaseH Plus) kit instructions. Reaction conditions: 95°C pre-denaturation 30 s; 95°C denatured 5 s, 58°C annealed 34 s, For 40 cycles. GAPDH was used as an internal standard, primers sequence used as follows:

Hoxd4-F: CTAGTCGCCGGCTGCGGGAT

Hoxd4-R: TTAGTCCCCCGGAGGGTGCG

GAPDH-F: 5′- ACGGATTTGGTCGTATTGGG -3′

GAPDH-R: 5′- TCATTTTGGAGGGATCTCGC -3′

After the reaction, the SDSShell software records the number of cycles that each hole reaches the set fluorescence threshold, the CT value. The average data of the HOXD4 was analyzed by Ct (Ct = Ct HOXD4-CtGAPDH). The smaller the Ct value predicted the higher expression of HOXD4.

### Immunohistochemical staining

All paraffin sections proceed with HE staining and locating in the first place, H_2_O_2_ was used to eliminate endogenous activity of catalase and rinsed by PBS, subsequently sections were incubated with antibody (HOXD4) at 4°C overnight, we predicted that when the tan particles appeared, coloration was determined. IHC staining was estimated by two independent investigators. Results can be divided into four grades according to the dyeing intensity staining: 0, negative; 1, weak; 2, moderate; and 3, strong, the percentage of immunostaining positive cells was also scored in four categories: 0 (0 %), 1 (1-33 %), 2 (34-66 %), and 3 (67-100 %). The final staining score was defined to be the summation of positive staining intensity score and cell percentage score. Finally a HOXD4 expression score ≥4 was regarded as high HOXD4 expression, meanwhile the score ≤3 was low HOXD4 expression.

### Statistical analysis

All data were tested for normality. The data of normal distribution were expressed as mean ± standard deviation, and two samples were used for *t* test. Mann-Whitney U test was used to compare the mRNA expression level. Fisher’s exact test was used to test possible associations between HOXD4 expression and clinicopathological feathers. Interval time from surgical treatment to the death or closest follow-up means overall survival (OS), meanwhile interval time from surgical treatment to the definite recurrence or closest follow-up means progression-free survival (PFS). Survival analysis curve was estimated using the Kaplan-Meier method, and appropriate variables were put into cox proportional hazards regression model to proceed multivariate analysis. When *p* value was less than 0.05, we admitted the statistical significance. GraphPad prism 5 (Graphpad Inc, La Jolla, USA) and IBM SPSS Statistics 23 (IBM Corp., Armonk, NY, USA) was used as data statistics software.

### Compliance with ethical standards

#### Informed consent

Informed consent was obtained from all individual participants included in the study.

#### Ethical approval

All procedures performed in studies involving human participants were in accordance with the ethical standards of the institutional research committee of the First Affiliated Hospital of Zhengzhou University.
